# Components of one-carbon metabolism and renal cell carcinoma: a systematic review and meta-analysis

**DOI:** 10.1007/s00394-020-02211-6

**Published:** 2020-03-11

**Authors:** Joanna L. Clasen, Alicia K. Heath, Ghislaine Scelo, David C. Muller

**Affiliations:** 1 Department of Epidemiology and Biostatistics, School of Public Health, Imperial College London, London, UK; 2 International Agency for Research on Cancer, Lyon, France; 3 Department of Epidemiology and Biostatistics, School of Public Health, MRC-PHE Centre for Environment and Health, Imperial College London, London, UK

**Keywords:** Renal cell carcinoma, Kidney cancer, One-carbon metabolism, Bayesian meta-analysis, Dietary biomarkers

## Abstract

**Purpose:**

Little is known about the aetiology of renal cell carcinoma (RCC). Components of one-carbon (1C) metabolism, which are required for nucleotide synthesis and methylation reactions, may be related to risk of RCC but existing evidence is inconclusive. We conducted a systematic review and independent exposure-specific meta-analyses of dietary intake and circulating biomarkers of 1C metabolites and RCC risk.

**Methods:**

Medline and Embase databases were searched for observational studies investigating RCC or kidney cancer incidence or mortality in relation to components of 1C metabolism and 12 eligible articles were included in the meta-analyses. We used Bayesian meta-analyses to estimate summary relative risks (RRs) and 95% credible intervals (CrIs) comparing the highest versus lowest categories as well as the between-study heterogeneity.

**Results:**

We did not find convincing evidence of an association between any exposure (riboflavin, vitamin B_6_, folate, vitamin B_12_, methionine, homocysteine, choline, or betaine) and RCC risk. However, vitamin B_6_ biomarker status did have a protective (RR = 0.62) but imprecise (95% CrI 0.39–1.14) effect estimate and folate intake had a notable association as well (RR = 0.85, 95% CrI 0.71–1.01).

**Conclusion:**

There was a lack of precision due largely to the low number of studies. Further investigation is warranted, especially for folate and vitamin B_6_, which had consistent suggestive evidence of a protective effect for both dietary intake and biomarker status. A unique strength of this review is the use of Bayesian meta-analyses which allowed for robust estimation of between-study heterogeneity.

**Electronic supplementary material:**

The online version of this article (10.1007/s00394-020-02211-6) contains supplementary material, which is available to authorized users.

## Introduction

Renal cell carcinoma (RCC) comprises 90% of kidney cancers in adults and is the 13th most common cause of cancer death globally [1, 2]. Relatively little is known about the aetiology of RCC, with age, sex, adiposity, cigarette smoking, and hypertension being the only established risk factors. There is some evidence that diets rich in fruits and vegetables may be associated with lower risk of RCC [3], but determining any specific role of dietary factors has been a more elusive task [1]. B vitamins are an enticing candidate for linking diet with RCC risk because of their involvement in one-carbon (1C) metabolism, which is a prerequisite to multiple processes relevant to carcinogenesis including DNA methylation and nucleotide synthesis [4]. 1C metabolism involves the coordination of the folate cycle and the methionine cycle to generate S-adenosylmethionine, a universal methyl donor. Required dietary inputs include folate, riboflavin, vitamin B_6_, vitamin B_12_, and methionine [5]. RCC is of particular interest because kidney is one of the few tissues in which betaine–homocysteine methyltransferase is produced and betaine or choline can be used as the methyl donor in lieu of folate [6]. Therefore, the role of 1C metabolism in RCC might differ from what has been observed for other cancers.

The aim of this review was to systematically examine the existing evidence on the association between components of 1C metabolism, both dietary intake and circulating biomarkers, and risk of RCC, and to present a quantitative summary of these relationships by conducting meta-analyses.

## Methods

### Study selection

Medline and Embase databases were searched on April 5th, 2019 for records of observational studies related to RCC or kidney cancer incidence or mortality and 1C metabolism. A medical school librarian was consulted for selection of search terms. The exposures were intake or circulating biomarkers of riboflavin (vitamin B_2_), vitamin B_6_, folate (vitamin B_9_), vitamin B_12_, methionine, homocysteine, choline, or betaine. The full search strategy for Medline is provided in Online Resource 1.

Results from the database searches were combined and duplicates were removed. Two investigators (JLC and AKH) independently screened the titles and abstracts with a web-based screening software and removed ineligible papers [7]. Papers were excluded if the exposure data did not represent status prior to diagnosis. The full texts of the remaining papers were read by both investigators and those meeting all eligibility criteria were included in the systematic review. The reference lists of all selected papers were searched for additional eligible records and a forward citation search was conducted to identify more recent papers that have cited the selected records. Where multiple publications used data from the same study with the same outcome of interest, the most recently published record was kept. Only papers examining individual nutrient exposures were included in the meta-analyses.

### Data extraction

Data collected for each paper included: author, publication year, study name and location, study design, number of cases, cohort size or number of controls, years of follow-up, sex distribution, age range, measured exposures of interest, outcome (RCC or all kidney cancer), covariates included in the most comprehensively adjusted model, effect estimate and 95% confidence interval (CI), and criteria for the Newcastle–Ottawa Scale (NOS) assessment. The summary measure of interest was the relative risk (RR); however, RCC is a sufficiently rare disease to also include odds ratios (ORs), hazard ratios (HRs), and standardised incidence ratios (SIRs) as reasonable approximations of the RR. If an article reported pooled results from more than one study, only the individual study results were used. All data were collected by one investigator (JLC) and verified by a second (AKH).

### Quality assessment

After consideration of several quality assessment tools, the NOS scale was selected as the most appropriate for use with observational studies [8]. In the NOS system, a maximum of 9 stars are awarded to each study, with stars deducted for suboptimal study design or reporting. Studies with fewer stars may be more prone to bias. We used a modified version of NOS which was tailored for use with dietary data and cancer outcomes and each study within each publication was rated separately. There are separate sets of questions for case–control versus cohort studies and nested case–control and case–cohort studies were rated on the cohort study version.

### Statistical analysis

Each exposure was modelled individually and dietary intake and biomarkers were assessed separately. All dietary components of 1C metabolism (riboflavin, vitamin B_6_, folate, vitamin B_12_, methionine, betaine, and choline) as well as homocysteine were analysed if data were available from at least two different studies (*k* ≥ 2). All effect estimates were log-transformed for analysis.

A Bayesian approach was used for the meta-analyses to estimate summary RRs and 95% credible intervals (CrIs) for the highest versus lowest category of the exposures. A CrI is analogous to a confidence interval in frequentist analysis, but it has the advantage of a more intuitive definition in that it represents the probability that the population parameter lies within the specified range conditional on the model and the data [9]. The key difference between Bayesian and random-effects frequentist analytical methods for meta-analysis is the estimation of the between-study variance, *τ*^2^. The random-effects frequentist approach treats the variance as a known quantity, whereas the Bayesian approach uses a distribution to represent the uncertainty in *τ*^2^. Similar results are usually obtained when *k* is large, but when the number of studies is small the estimate of τ^2^ lacks precision, and results from the frequentist method are less reliable in this case [10]. Since the results of Bayesian analyses depend on both the prior and the likelihood, it is important to consider a range of prior distributions and assess their impact on the posterior distribution. Therefore, a series of priors were used in this analysis, selected on the basis of theoretical and empirical reasoning. For the main model, the distribution for *µ* (the log relative risk, in this case) is normal with a mean of 0 and standard deviation of 0.82 and the distribution for *τ* (the between-study standard deviation) is log-normal with a log mean of -3.27 and log standard deviation of 1.68. The distribution for µ is derived from an assumption that a relative risk of 5 is a generous upper limit for the expected estimated effect and a standard deviation of 0.82 allows for 95% of the distribution to be as or less extreme compared to this limit, assuming a normal distribution. We consider this a “weakly informative” prior distribution, in that it puts low probability on implausible parameter values, and substantial probability over the range of plausible parameter values. The distribution for τ is suggested by Turner et al. for use in a meta-analysis of a “major morbidity event” comparing non-pharmacological exposures [11]. The prior was derived based on observed heterogeneity in binary outcome meta-analyses from the Cochrane Database of Systematic Reviews. This method allows for incorporation of prior knowledge to refine the estimated heterogeneity.

Two other Bayesian models were run to assess sensitivity of the results to the prior distribution. The first uses the same distribution for *µ* and a half-normal distribution for *τ*, which has been shown to be an appropriate distribution in meta-analyses with a small number of studies [12]. The second employs a very weak prior for both *µ* and *τ* and it is intended to test the impact of extreme alteration of prior distributions. We avoided using a uniform prior because placing equal density across all real values would give too much weight to implausible values. Frequentist random-effects and fixed-effect models were run as well. The restricted maximum likelihood estimator was used in the frequentist random-effects models.

The primary outcome of interest was RCC. Because a majority of kidney cancers in adults are RCC, studies assessing risk of overall kidney cancer were included in a secondary analysis to increase the number of studies available.

Multiple sensitivity analyses, specified a priori, were undertaken to assess the robustness of the results. Studies with a lower NOS score are assumed to be more susceptible to bias, so those with a NOS score less than seven were excluded for the first sensitivity analysis. For the next sensitivity analysis, all case–control studies were excluded for dietary intake exposures to avoid the risk of recall bias from retrospectively collected data. Finally, we conducted analyses on dietary intake exposures for food consumption only, excluding supplement use, because of the differences in bioavailability and data collection methods.

Funnel plots were visually inspected to check the risk of publication bias and heterogeneity was assessed with *τ*^2^ and *I*^2^ based on the posterior distribution of *τ*. *I*^2^ is an intuitive measure of heterogeneity, indicating the percentage of total variation in the estimated associations due to between-study heterogeneity. We have also reported *τ*^2^, indicating the between-study variance because, unlike *I*^2^, it is not dependent on the number or size of included studies [13].

Meta-analyses were done using R 3.6.0 [14], the bayesmeta package [15], and the metafor package [16].

## Results

### Literature search

**Fig. 1:**
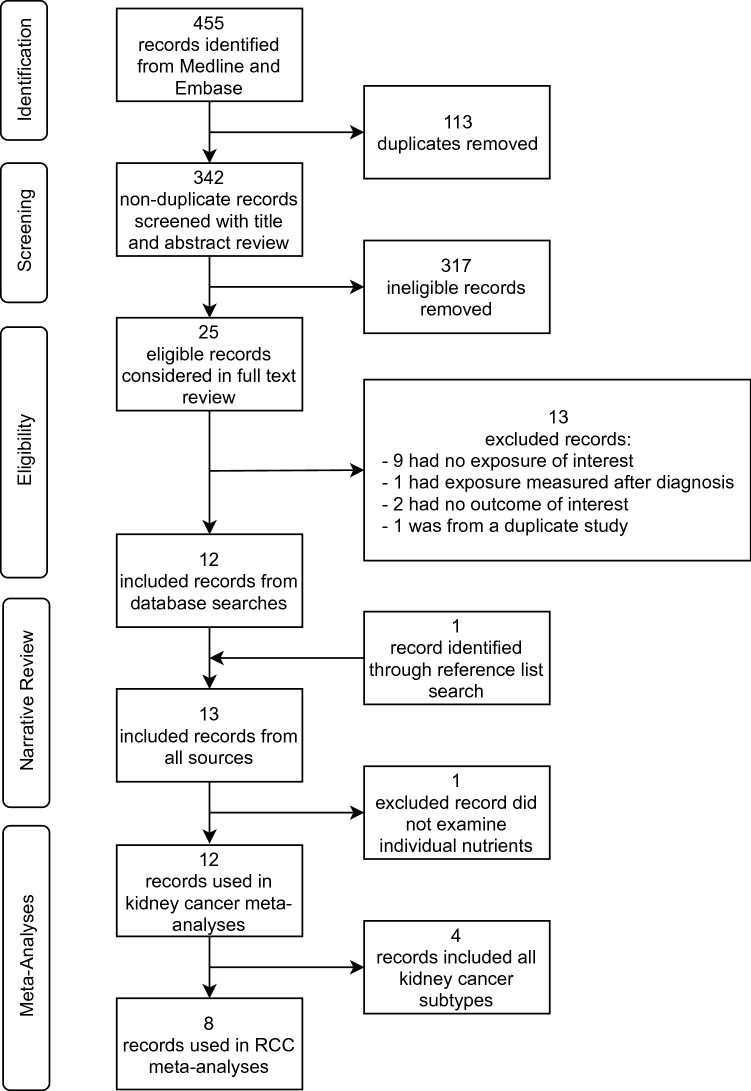
Flow chart of study selection for the systematic review and meta-analysis of components of 1C metabolism and RCC risk

Figure 1 shows the results of the database search and study selection. The database searches produced 455 total records and 113 duplicates were removed, leaving 342 unique records. After title and abstract review, 317 records were deemed ineligible, leaving 25 records for full-text review. Of these 25, 9 were excluded for no exposure of interest, 1 was excluded for exposure measured after diagnosis, 2 were excluded for no outcome of interest, and 1 was excluded due to multiple publications from the same study population. The 12 remaining records were included in the review. Additionally, the references and forward citations of these 12 records were searched and 1 additional record was identified. Therefore, a total of 13 records were included in the systematic review. One of these papers examined B-complex vitamins rather than individual nutrients and was excluded from the meta-analyses, therefore 12 records were included in the meta-analyses. Of these 12, 8 examined incident RCC risk and 4 examined risk for any type of kidney cancer. Two of the eight RCC outcome publications included data from two studies each, so a total of ten studies were included in the RCC meta-analyses. Of the ten studies, three included riboflavin intake, five included vitamin B_6_ intake, six included folate intake, three included vitamin B_12_ intake, two included methionine intake, two included choline intake, and two included betaine intake. For biomarkers, analyses from the same two studies, the European Prospective Investigation into Cancer and Nutrition (EPIC) and the Alpha-Tocopherol Beta-Carotene Cancer Prevention Study (ATBC), included riboflavin, folate, vitamin B_12_, and homocysteine, and these two studies plus the Melbourne Collaborative Cohort Study (MCCS) included vitamin B_6_. There was a mean of 2.8 studies per exposure. There were not enough studies examining mortality to be able to perform meta-analysis on this outcome.

### Study characteristics

The 13 papers were published between 1997 and 2018 and they included a total of 13 different study populations (Table 1) [17–29]. Five of these studies were conducted in Europe, six were in North America, one in Australia, and one in South America. Two studies were restricted to women only, two restricted to men only, and the remaining nine enrolled both sexes.

**Table 1:** Systematic review study characteristics

Author and year	Study name and location	Study design	Cases	Total *N* (cohorts) or controls or sub-cohort	Years of follow-up	Sex (% men)	Age range (at baseline for cohorts)	Exposures measured	Outcome	Adjusted covariates	Statistic	NOS score
Arendt 2013	Denmark	Cohort	445	333,667	Median 3.5	41%	IQR 40.2–69.2	Plasma B12	Kidney	Age, sex, calendar year	SIR	4.0
Aune 2010	Uruguay	Case–control	114	2032	N/A	66%	23–89 (cases) 22–89 (controls)	Dietary B9	Kidney	Age, sex, BMI, smoking, alcohol, energy intake, calcium, iron, fibre, mate drinking, intake of vitamins B6, C, and E, and carotenoids, education, income, urban/rural residence, interviewer	OR	6.0
Bock 2018	The US Kidney Cancer Study (USA)	Case–control	1142	1154	N/A	55%	20–79	Dietary B1, B2, B3, B6, B9, B12	RCC	Age, sex, BMI, smoking, alcohol, energy intake, hypertension, family history, education, region, race	OR	6.0
Bosetti 2006	Italy	Case–control	767	1534	N/A	64%	24–79 (cases) 22–79 (controls)	Dietary B1, B2, B3, B6, B9	RCC	Age, sex, BMI, smoking, alcohol, family history, education, centre, period of interview	OR	8.0
Brock 2012	Iowa, USA	Case–control	323	1827	N/A	67%	40–85	dietary B9	RCC	Age, sex, BMI at age 40, smoking, alcohol, energy intake, fatty spreads consumption, hypertension, proxy status	OR	7.0
Cho 2013	NHS (USA)	Cohort	225	77,208	24	0%	30–55	Total intake B6, B9, B12, methionine	RCC	Age, BMI, smoking, alcohol, energy intake, fruit and vegetable consumption, hypertension, diabetes, parity calendar time	RR	5.5
Cho 2013	HPFS (USA)	Cohort	211	47,886	22	100%	40–75	Total intake B6, B9, B12, methionine	RCC	Age, BMI, smoking, alcohol, energy intake, fruit and vegetable consumption, hypertension, diabetes, calendar time	RR	5.5
Gibson 2010	ATBC (Finland)	Nested case–control	224	Not reported	Not reported	100%	50–69	Serum B2, B6, B9 B12, homocysteine	RCC	All nutrients: age, BMI and smoking; B9 only: protein and fat intake; B6, B2, and homocysteine only: serum B9; B12 only: protein, leisure time physical activity and serum B9; alcohol checked but not included as not found to be a confounder	OR	7.0
Hu 2003	NECSS (Canada)	Case–control	1110	4708	N/A	51%	20–70+	B-complex supplements	RCC	Age, BMI, smoking, alcohol, education, province	OR	7.0
Johansson 2014	EPIC (Europe)	Nested case–control	556	556 matched; 553 unmatched	Not reported	Matched 56%; unmatched 68%	Not reported	Plasma B2, B6, B9, B12, methionine, homocysteine	RCC	Age, sex, waist-to-hip ratio, smoking, plasma cotinine, alcohol, hypertension, education, country	OR	8.5
Johansson 2014	MCCS (Australia)	Nested case–control	144	144	Not reported	Both, % men not specified	40–79	Plasma B6	RCC	Age, sex, waist-to-hip ratio, smoking, plasma cotinine, alcohol, hypertension, education, country	OR	9.0
Nicodemus 2004	Iowa Women's Health Study (USA)	Cohort	124	34,637	15	0%	55–69	Dietary B1, B2, B6	Kidney	Age	RR	5.0
Prineas 1997	Iowa Women's Health Study (USA)	Cohort	62	35,192	8	0%	55–69	Dietary B1, B2, B6	RCC	Age	RR	4.0
Schouten 2016	NLCS (The Netherlands)	Case–cohort	498	3980	20.3	49%	55–69	Dietary B9	RCC	Age, sex, BMI, smoking, alcohol, energy intake, intake of methionine and B2 and B6, hypertension	HR	8.0
Tavani 2012	Italy	Case–control	767	1534	N/A	64%	Not reported	Dietary B9	Kidney	Age, sex, BMI, smoking, alcohol, energy intake, education, study centre, year of interview, physical activity at work	OR	5.0

NHS, Nurses’ Health Study; HPFS, The Health Professionals Follow-up Study; ATBC, Alpha-Tocopherol Beta-Carotene Cancer Prevention Study; NECSS, National Enhanced Cancer Surveillance System; EPIC, European Prospective Investigation into Cancer and Nutrition; MCCS, Melbourne Collaborative Cohort Study; NLCS, The Netherlands Cohort Study on Diet and Cancer

### Quality assessment

Out of a maximum of nine stars, study quality assessment scores from the modified NOS scale ranged from five to eight for case–control studies and four to nine for cohort studies (including nested case–control and case–cohort studies). The mean scores were 6.5 for case–control studies and 6.3 for cohort studies. The lowest scoring topics were ascertainment of exposure in case–control studies and ascertainment of exposure plus representativeness of the cohort for cohort studies.

### Meta-analyses

**Fig. 2:**
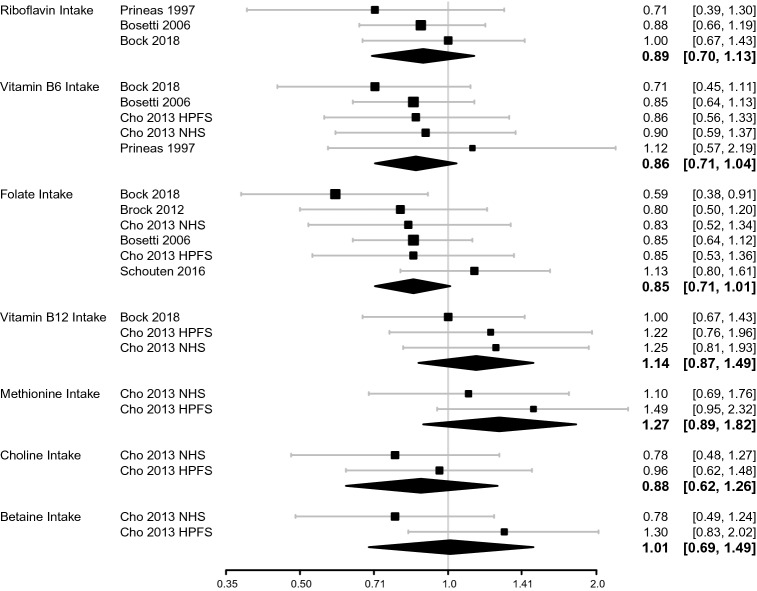
Forest plots for 1C metabolism dietary intake exposures in relation to RCC risk. Diamonds represent the pooled RR and 95% CrI

Results for the meta-analyses of intake are presented in Fig. 2. There was suggestion of a protective association for intakes of riboflavin, vitamin B_6_, folate and choline, but estimates were accompanied by substantial uncertainty, particularly for riboflavin and choline. The direction of effect estimates varied between nutrients, with vitamin B_12_, methionine, and betaine having relative risks greater than 1. The pooled estimates for highest versus lowest category were as follows for intake exposure: RR = 0.89 (95% CrI 0.70–1.13) for riboflavin, RR = 0.86 (95% CrI 0.71–1.04) for vitamin B_6_, RR = 0.85 (95% CrI 0.71–1.01) for folate, RR = 1.14 (95% CrI 0.87–1.49) for vitamin B_12_, RR = 1.27 (95% CrI 0.89–1.82) for methionine, RR = 0.88 (95% CrI 0.62–1.26) for choline, and RR = 1.01 (95% CrI 0.69–1.49) for betaine.

**Fig. 3:**
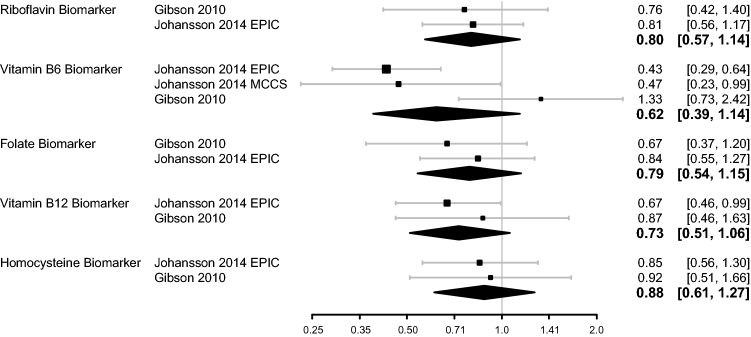
Forest plots for 1C metabolism circulating biomarker exposures in relation to RCC risk. Diamonds represent the pooled RR and 95% CrI

Figure 3 shows results of the meta-analyses of biomarkers. The pooled estimates for highest versus lowest concentration category for biomarkers were as follows: RR = 0.80 (95% CrI 0.57–1.14) for riboflavin, RR = 0.62 (95% CrI 0.39–1.14) for vitamin B_6_, RR = 0.79 (95% CrI 0.54–1.15) for folate, RR = 0.73 (95% CrI 0.51–1.06) for vitamin B_12_, and RR = 0.88 (95% CrI 0.61–1.27) for homocysteine. All biomarkers showed estimates in the direction of a protective effect, however, the estimates were lacking precision. No papers examined biomarker status for choline or betaine and only one had results for methionine biomarker status, hence these three exposures could not be included in the meta-analyses.

Visual inspection of funnel plots for intake and biomarkers did not indicate publication bias. However, the low number of included primary studies for most exposures precludes any reliable assessment of publication bias. Posterior distributions for *τ* indicated a lack of between-study heterogeneity for all exposures except vitamin B_6_ biomarker status (Table 2).

**Table 2:** Between-study heterogeneity for the Bayesian and frequentist random-effects models

Exposure	Bayesian (values based on the posterior distribution of *τ*)		Frequentist random-effects	
	*τ*^2^ (95% CrI)	I^2^ (95% CrI)	*τ*^2^	*I*^2^
Dietary intake				
Riboflavin	0.001 (0–0.046)	1.7 (0–51.0)	0.000	0.000
Vitamin B_6_	0.001 (0–0.023)	1.2 (0–33.6)	0.000	0.000
Folate	0.001 (0–0.039)	2.1 (0–49.1)	0.007	15.360
Vitamin B_12_	0.001 (0–0.046)	1.5 (0–48.0)	0.000	0.000
Methionine	0.001 (0–0.1)	1.9 (0–64.6)	0.000	0.000
Choline	0.001 (0–0.084)	1.7 (0–60.1)	0.000	0.000
Betaine	0.002 (0–0.18)	2.9 (0–76.8)	0.076	58.357
Biomarker status				
Riboflavin	0.001 (0–0.077)	1.4 (0–54.8)	0.000	0.000
Vitamin B_6_	0.047 (0–0.807)	37.0 (0–90.9)	0.312	79.384
Folate	0.001 (0–0.09)	1.4 (0–56.4)	0.000	0.000
Vitamin B_12_	0.001 (0–0.097)	1.4 (0–57.7)	0.000	0.000
Homocysteine	0.001 (0–0.079)	1.3 (0–53.9)	0.000	0.000

The Bayesian method allows for uncertainty in the estimation of heterogeneity and therefore 95% CrIs are reported for the Bayesian model parameters

## Sensitivity analyses

Overall, the frequentist random-effects models gave similar results to those from the main models (Online Resources 2–3). Vitamin B_12_ biomarker status had a similar estimate but greater precision with the frequentist random-effects model (RR = 0.72, 95% CI 0.52–0.99). Estimates of between-study heterogeneity were less extreme for the main Bayesian model compared to the frequentist random-effects estimates which ranged from *I*^2^ = 0% for 9 exposure to *I*^2^ = 79.4% for vitamin B_6_ biomarker status (Table 2). Bayesian estimates for τ were higher only when the frequentist random-effects estimate was τ = 0 (Online Resources 4–5).

Frequentist fixed-effect model results differed from the random-effects models only for exposure where the estimate of *I*^2^ was greater than 0%, namely folate intake and vitamin B_6_ biomarker status. The effect estimate for folate intake from the fixed-effect model compared to the random-effects model was identical with a slightly more precise CI (RR = 0.85, 95% CI 0.72–0.99) and the estimate for vitamin B_6_ biomarker status was slightly stronger (RR = 0.58, 95% CI 0.43–0.78) in the fixed-effect model.

There were no notable differences when analyses were run with the other two Bayesian models (Online Resources 6–9). The point estimates were substantively and qualitatively unchanged for all exposures and the 95% CrIs were slightly wider for the first alternate Bayesian model and much wider for the second alternate Bayesian model.

We also conducted an analysis with any kidney cancer as the outcome rather than RCC. Two publications reporting kidney cancers replaced two older publications from the same studies reporting RCC only (Nicodemus 2004 replaced Prineas 1997 and Tavani 2012 replaced Bosetti 2006). Two additional kidney cancer publications were added, so a total of ten studies were available for analyses. Meta-analyses were possible for only three exposures: riboflavin intake, folate intake, and vitamin B_12_ biomarker concentration (Online Resource 10). There was not a considerable change for riboflavin intake (RR = 0.89, 95% CrI 0.70–1.14) or folate intake (RR = 0.83, 95% CrI 0.70–0.998) compared to the RCC-only results. The pooled estimate for vitamin B_12_ biomarker status changed most substantially, from RR = 0.73 (95% CrI 0.51–1.06) to RR = 1.07 (95% CrI 0.55–2.24), and there was considerable heterogeneity between primary studies (*I*^2^ = 75% for the posterior median of *τ*).

When including only studies with a NOS score of seven or greater, only folate intake met the criteria of having a different set of two or more studies available compared to those included in the original analysis (Online Resource 11). The pooled estimate was marginally weaker than that from the main analysis (RR = 0.93, 95% CrI 0.74–1.16, based on three studies). Exposures already having only studies with a NOS score of at least seven in the original analyses were riboflavin biomarker status, vitamin B_6_ biomarker status, folate biomarker status, vitamin B_12_ biomarker status, and homocysteine biomarker status.

Four studies examining dietary intake were included after removing case–control studies (Online Resource 12). For vitamin B_6_ intake, the two most protective estimates were from case–control studies in the original model, so the pooled estimate was weaker when these were dropped (RR = 0.92, 95% CrI 0.69–1.23). Similarly, three of the four most extreme estimates for folate intake were removed and the resulting pooled estimate was weaker (RR = 0.97, 95% CrI 0.74–1.26). One case–control study was removed for vitamin B_12_ intake, leaving only the two estimates from the Cho 2013 paper. Our pooled result (RR = 1.22, 95% CrI 0.86–1.73) was similar to that presented by Cho et al. (RR = 1.24, 95% CI 0.90–1.70) [22]. Unlike the other two exposures analysed without case–control studies, the pooled estimate for vitamin B_12_ intake was stronger than from our main analysis.

For estimates of food intake only, excluding supplements, riboflavin (RR = 0.92, 95% CrI 0.70–1.21), vitamin B_6_ (RR = 0.88, 95% CrI 0.71–1.10), and folate (RR = 0.90, 95% CrI 0.75–1.09) all had a slightly weaker pooled estimate compared to the original models (Online Resource 13). The estimate for vitamin B_12_ intake from food was slightly stronger than that from the main analysis, but still accompanied by substantial uncertainty (RR = 1.21, 95% CrI 0.92–1.60).

## Discussion

The purpose of this systematic review and meta-analysis was to assess the existing evidence for an association between 1C metabolites and RCC risk. None of the individual intake or biomarker exposures analysed had a pooled estimate with enough precision to indicate a clear association with RCC risk. This lack of precision is largely attributable to the low number of studies and in some cases to between-study heterogeneity. There was a range of two to six studies per exposure, and additional exposures of interest were left out because they had fewer than two studies available. Areas warranting further investigation were identified, including the three B vitamins involved in the folate cycle (riboflavin, vitamin B_6_, and folate), which all had pooled estimates suggestive of a protective association. This advocates for a role by nucleotide synthesis in a mechanistic explanation of association because of its direct link with the folate cycle.

There were four metabolites which were included in both the intake and biomarker analyses (riboflavin, vitamin B_6_, folate, and vitamin B_12_) and each of these had a stronger pooled RR for biomarker status over intake. There are multiple factors that affect the link from intake to circulating concentration of a nutrient including biosynthesis by the microbiome, variable rates of absorption and de novo synthesis regulated by feedback mechanisms, uneven distribution between tissues and in circulation, and measurement error, particularly in the measurement of dietary intake. Vitamin B_12_ was the only nutrient with a qualitative difference between intake (RR greater than 1) and biomarker status (RR less than 1). Unlike the RCC-specific estimate, the biomarker estimate including any kidney cancer (RR = 1.07) was in the same direction as the intake estimate; however, there is considerable between-study heterogeneity for this estimate largely driven by the Arendt 2013 study. This study differed from the others in that the median follow-up time after blood draw was only 3.5 years, so their results may be more strongly affected by reverse causation.

Three exposures (methionine, choline, and betaine) only included data from the NHS and HPFS studies within the Cho 2013 paper, which also presented pooled results from the two studies. The estimates found from our model were very similar to those presented by Cho et al. [22].

Our analysis included three more RCC-specific studies than the most recently published meta-analysis, and also assessed additional key 1C metabolites choline and betaine. Overall, our results are similar to those from the previous meta-analysis, which found no clear associations with RCC for any of the 1C metabolism intake or biomarker exposures examined, though it did provide weak evidence for a protective effect of vitamin B_12_ biomarker status [30]. The dose–response portion of their meta-analysis determined that riboflavin, vitamin B_6_, and vitamin B_12_ biomarker statuses are inversely associated with RCC risk. Whilst we did not complete a dose–response analysis because of differences in intake adjustment methods and biomarker measurement methods between studies, our results are broadly consistent with those previously published.

1C metabolism may play a parallel role in kidney cancer and liver cancer, because these are the only two organs where betaine can be used rather than only folate as the methyl donor [6]. Research from the ATBC study found no association of any 1C metabolite and risk of liver cancer, which is consistent with the lack of clear association found here for RCC risk [31]. Other tumour sites have been investigated for association with 1C metabolism as well. A meta-analysis on lung cancer risk found evidence of a protective association for higher circulating folate and vitamin B_6_ and increased risk for higher circulating homocysteine [32]. This meta-analysis included more primary studies than ours and therefore reported more precise pooled estimates. One-carbon metabolites have also been extensively studied in relation to colorectal cancer, but there is no strong consensus on their relationships with risk. A meta-analysis found no association for folic acid supplement use or red blood cell folate status and colorectal cancer risk, but it did find an inverse association with total folate intake [33]. In contrast, prostate cancer was shown in a meta-analysis to have a higher risk with increasing vitamin B_12_ concentration and the evidence suggests a positive association with circulating folate as well [34]. Folate intake is of particular interest in part because of these and other previously reported divergent associations with cancer risk [35]. Our results suggest a protective role for folate against RCC, but further research is needed to assess the linearity of this association and to investigate potential causal mechanisms.

A major risk of bias in meta-analyses comes from selective reporting of results within studies. Some included papers stated that all measured associations were selected a priori, but in other papers it is not clear if there may have been bias in the selection of reported results. The Nicodemus 2004 paper stated that no association was found for folic acid supplementation or vitamin B_6_ intake, but a specific RR was not given so these exposures could not be included in meta-analyses. We did not attempt to contact authors to obtain data not presented in the papers included in this review.

One limitation of this meta-analysis is the inconsistency of covariates included in the primary analyses. Some models were only adjusted for basic demographics and did not account for key established risk factors for RCC including sex and body mass index. Some primary analyses in our meta-analysis did not adjust for concurrent nutrient intakes, while those that did are likely affected by residual confounding from unidentified nutritional components.

Population diversity is lacking in the studies included in this meta-analysis, with a strong overrepresentation of participants of European descent, largely from affluent countries. Bock et al. did compare European-American and African-American participants and found similar associations for the two groups [19]. The Aune 2010 study is the only one from a Latin American country and its relatively low OR for folate intake suggests the presence of heterogeneity across populations [18], but further studies in diverse populations are required to examine this possibility. As indicated by Aune et al., the low average folate intake in the study likely allowed for a clearer look at associations with folate deficiency.

A strength of this meta-analysis was the use of a Bayesian model complemented by several additional models to assess the impact of implied assumptions and our choice of priors. This allowed for a more robust estimation of the between-study heterogeneity compared to using frequentist methods as well as the propagation of uncertainty through to the effect estimates. This is especially important when the number of studies is small. Further, we conducted relevant sensitivity analyses, chosen a priori, to check the robustness of our results against common sources of bias.

Because tumours as well as healthy cells rely on 1C metabolism, the related risk factors for RCC prognosis may differ from those for RCC incidence. Unfortunately, despite their inclusion in the literature search, prognostic outcomes could not be included in the meta-analysis due to a lack of existing publications. This largely unexplored area will become increasingly important following predictions of a rising number of RCC cases [36].

In summary, the results of this systematic review and meta-analysis do not provide overwhelming evidence for the role of any single component of 1C metabolism in RCC risk, but the findings are based on sparse data. This is consistent with an overall lack of consensus on the role of 1C metabolism in multiple types of cancer. The suggestive evidence of inverse associations for both intake and circulating concentrations of several 1C metabolism components warrant further investigation.

## Electronic supplementary material

Below is the link to the electronic supplementary material.

Supplementary file1 (PDF 843 kb)

## References

[1] Chow W-H, Dong LM, Devesa SS (2010). Epidemiology and risk factors for kidney cancer. Nat Rev Urol.

[2] Capitanio U, Bensalah K, Bex A (2019). Epidemiology of renal cell carcinoma. Eur Urol.

[3] Lee JE, Männistö S, Spiegelman D (2009). Intakes of fruit, vegetables, and carotenoids and renal cell cancer risk: a pooled analysis of 13 prospective studies. Cancer Epidemiol Biomarkers Prev.

[4] Newman AC, Maddocks ODK (2017). One-carbon metabolism in cancer. Br J Cancer.

[5] Anderson OS, Sant KE, Dolinoy DC (2012). Nutrition and epigenetics: an interplay of dietary methyl donors, one-carbon metabolism and DNA methylation. J Nutr Biochem.

[6] Ducker GS, Rabinowitz JD (2017). One-Carbon metabolism in health and disease. Cell Metab.

[7] Ouzzani M, Hammady H, Fedorowicz Z, Elmagarmid A (2016) Rayyan QCRI, the systematic reviews web app. https://rayyan.qcri.org/welcome. Accessed 14 May 2019 10.1186/s13643-016-0384-4 PMC5139140 27919275

[8] Wells G, Shea B, O’Connell D et al (2019) The Newcastle-Ottawa Scale (NOS) for assessing the quality of nonrandomised studies in meta-analyses. https://www.ohri.ca/programs/clinical_epidemiology/oxford.asp. Accessed 14 May 2019

[9] Lambert B (2018). A student’s guide to Bayesian statistics.

[10] Pullenayegum EM (2011). An informed reference prior for between-study heterogeneity in meta-analyses of binary outcomes. Stat Med.

[11] Turner RM, Jackson D, Wei Y (2014). Predictive distributions for between-study heterogeneity and simple methods for their application in Bayesian meta-analysis. Statist Med.

[12] Friede T, Röver C, Wandel S, Neuenschwander B (2017). Meta-analysis of few small studies in orphan diseases. Res Synth Methods.

[13] Rücker G, Schwarzer G, Carpenter JR, Schumacher M (2008). Undue reliance on I(2) in assessing heterogeneity may mislead. BMC Med Res Methodol.

[14] R Core Team (2019). R: a language and environment for statistical computing.

[15] Roever C (2017) Bayesian random-effects meta-analysis using the bayesmeta R package. arXiv preprint arXiv:1711.08683

[16] Viechtbauer W (2010). Conducting meta-analyses in R with the metafor package. J Stat Softw.

[17] Arendt JFB, Pedersen L, Nexo E, Sørensen HT (2013). Elevated plasma vitamin B12 levels as a marker for cancer: a population-based cohort study. J Natl Cancer Inst.

[18] Aune D, Deneo-Pellegrini H, Ronco AL (2011). Dietary folate intake and the risk of 11 types of cancer: a case–control study in Uruguay. Ann Oncol.

[19] Bock CH, Ruterbusch JJ, Holowatyj AN (2018). Renal cell carcinoma risk associated with lower intake of micronutrients. Cancer Med.

[20] Bosetti C, Scotti L, Maso LD (2007). Micronutrients and the risk of renal cell cancer: A case-control study from Italy. Int J Cancer.

[21] Brock KE, Ke L, Gridley G (2012). Fruit, vegetables, fibre and micronutrients and risk of US renal cell carcinoma. Br J Nutr.

[22] Cho E, Giovannucci EL, Joh H-K (2013). Nutrients related to one-carbon metabolism and risk of renal cell cancer. Cancer Causes Control.

[23] Gibson TM, Weinstein SJ, Mayne ST (2010). A prospective study of one-carbon metabolism biomarkers and risk of renal cell carcinoma. Cancer Causes Control.

[24] Hu J, Mao Y, White K, Canadian Cancer Registries Epidemiology Research Group (2003). Diet and vitamin or mineral supplements and risk of renal cell carcinoma in Canada. Cancer Causes Control.

[25] Johansson M, Fanidi A, Muller DC (2014). Circulating biomarkers of one-carbon metabolism in relation to renal cell carcinoma incidence and survival. JNCI J Natl Cancer Inst.

[26] Nicodemus KK, Sweeney C, Folsom AR (2004). Evaluation of dietary, medical and lifestyle risk factors for incident kidney cancer in postmenopausal women. Int J Cancer.

[27] Prineas RJ, Folsom AR, Zhang M (1997). Nutrition and Other Risk Factors for Renal Cell Carcinoma in Postmenopausal Women. Epidemiology.

[28] Schouten LJ, Deckers IAG, van den Brandt PA (2016). Alcohol and Dietary folate intake and promoter CpG Island methylation in clear-cell renal cell cancer. Nutr Cancer.

[29] Tavani A, Malerba S, Pelucchi C (2012). Dietary folates and cancer risk in a network of case-control studies. Ann Oncol.

[30] Mao B, Li Y, Zhang Z (2015). One-carbon metabolic factors and risk of renal cell cancer: a meta-analysis. PLoS ONE.

[31] Schwartz LM, Persson EC, Weinstein SJ (2013). Alcohol consumption, one-carbon metabolites, liver cancer and liver disease mortality. PLoS ONE.

[32] Yang J, Li H, Deng H, Wang Z (2018). Association of one-carbon metabolism-related vitamins (Folate, B6, B12), homocysteine and methionine with the risk of lung cancer: systematic review and meta-analysis. Front Oncol.

[33] Moazzen S, Dolatkhah R, Tabrizi JS (2018). Folic acid intake and folate status and colorectal cancer risk: a systematic review and meta-analysis. Clin Nutr.

[34] Collin SM, Metcalfe C, Refsum H (2010). Circulating folate, vitamin B12, homocysteine, vitamin B12 transport proteins, and risk of prostate cancer: a case-control study, systematic review, and meta-analysis. Cancer Epidemiol Biomarkers Prev.

[35] Pieroth R, Paver S, Day S, Lammersfeld C (2018). Folate and its impact on cancer risk. Curr Nutr Rep.

[36] Wong MCS, Goggins WB, Yip BHK (2017). Incidence and mortality of kidney cancer: temporal patterns and global trends in 39 countries. Sci Rep.

