# Supplementary material for: Components of one-carbon metabolism and renal cell carcinoma: a systematic review and meta-analysis
Source: Eur J Nutr. 2020 Mar 11;59(8):3801–13. doi: 10.1007/s00394-020-02211-6 (PMC7669778; doi:10.1007/s00394-020-02211-6)
Supplement: Supplementary file 1 — Supplementary file1 (PDF 843 kb) [file 394_2020_2211_MOESM1_ESM.pdf]

Online Resource 1: Search used for the Ovid Medline database

1	Kidney Neoplasms/
2	Carcinoma, Renal Cell/
3	((renal or kidney or nephr*) adj3 (cancer* or tumo?r* or carcinoma* or neoplasm* or adenocarcinoma* or malignanc*)).mp.
4	1 or 2 or 3
5	exp Cohort Studies/
6	cohort*.tw.
7	controlled clinical trial.pt.
8	Epidemiologic Methods/
9	limit 8 to yr=1966-1989
10	exp case-control studies/
11	(case$ and control$).tw.
12	or/5-7,9-11
13	one-carbon metabolism.mp.
14	exp Vitamin B 6/
15	(b6 or b-6 or pyridox*).mp.
16	exp Folic Acid/
17	(folate or folic acid or tetrahydrofolate or b9 or b-9).mp.
18	exp Vitamin B 12/
19	(b12 or b-12 or cobalamin or cyanocobalamin).mp.
20	exp Riboflavin/
21	(b2 or b-2 or riboflavin).mp.
22	Betaine/

23	betaine.mp.
24	Choline/
25	choline.mp.
26	S-Adenosylhomocysteine/ or Homocysteine/
27	(homocysteine or s-adenosylhomocysteine).mp.
28	methionine/ or s-adenosylmethionine/
29	(methionine or s-adenosylmethionine or s-adenosyl methionine).mp.
30	*Diet/
31	"diet*".m_titl.
32	Vitamins/
33	"vitamin*".m_titl.
34	or/13-33
35	4 and 12 and 34

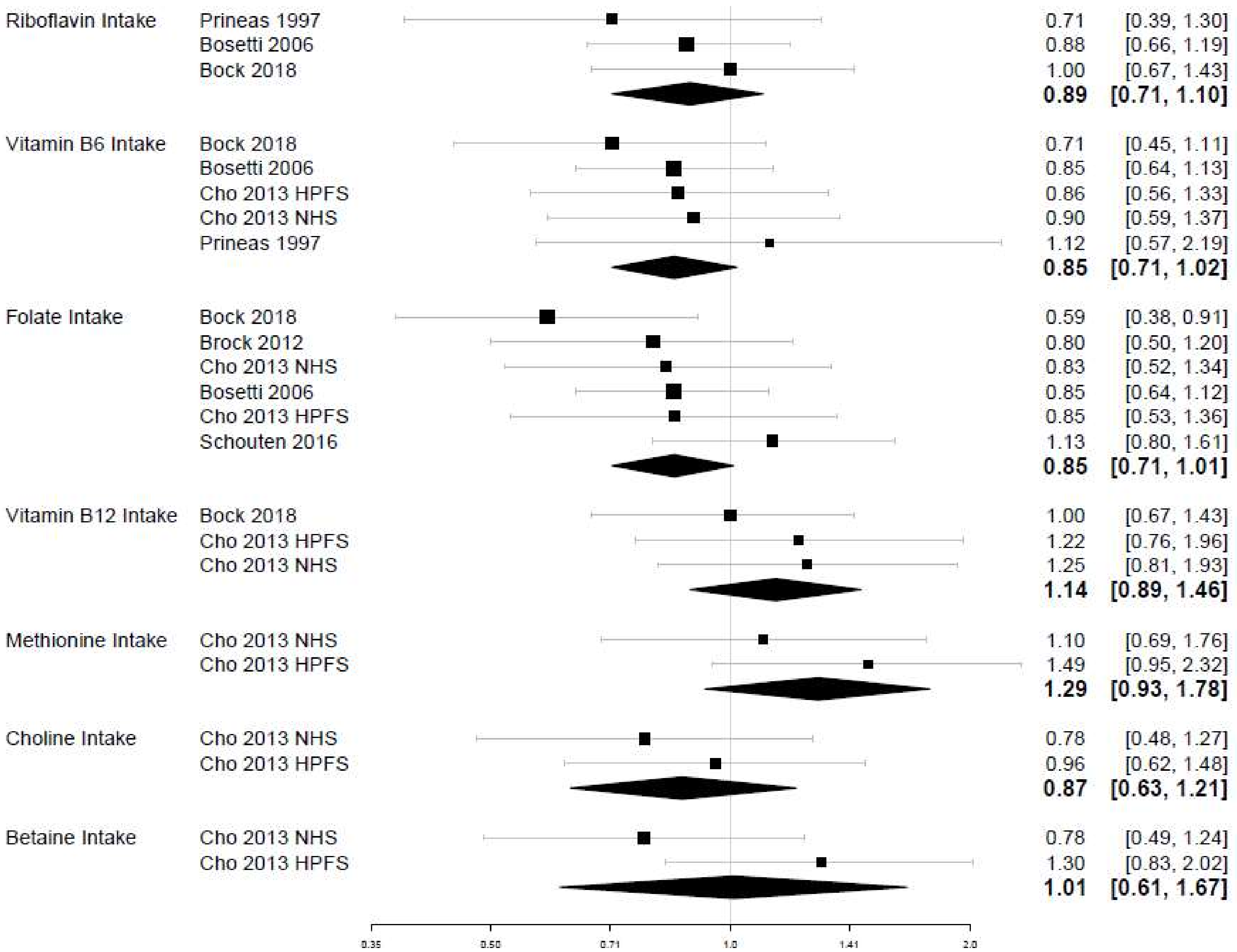

Online Resource 2: Forest plots for dietary intake exposures and RCC risk using a frequentist random-effects model.

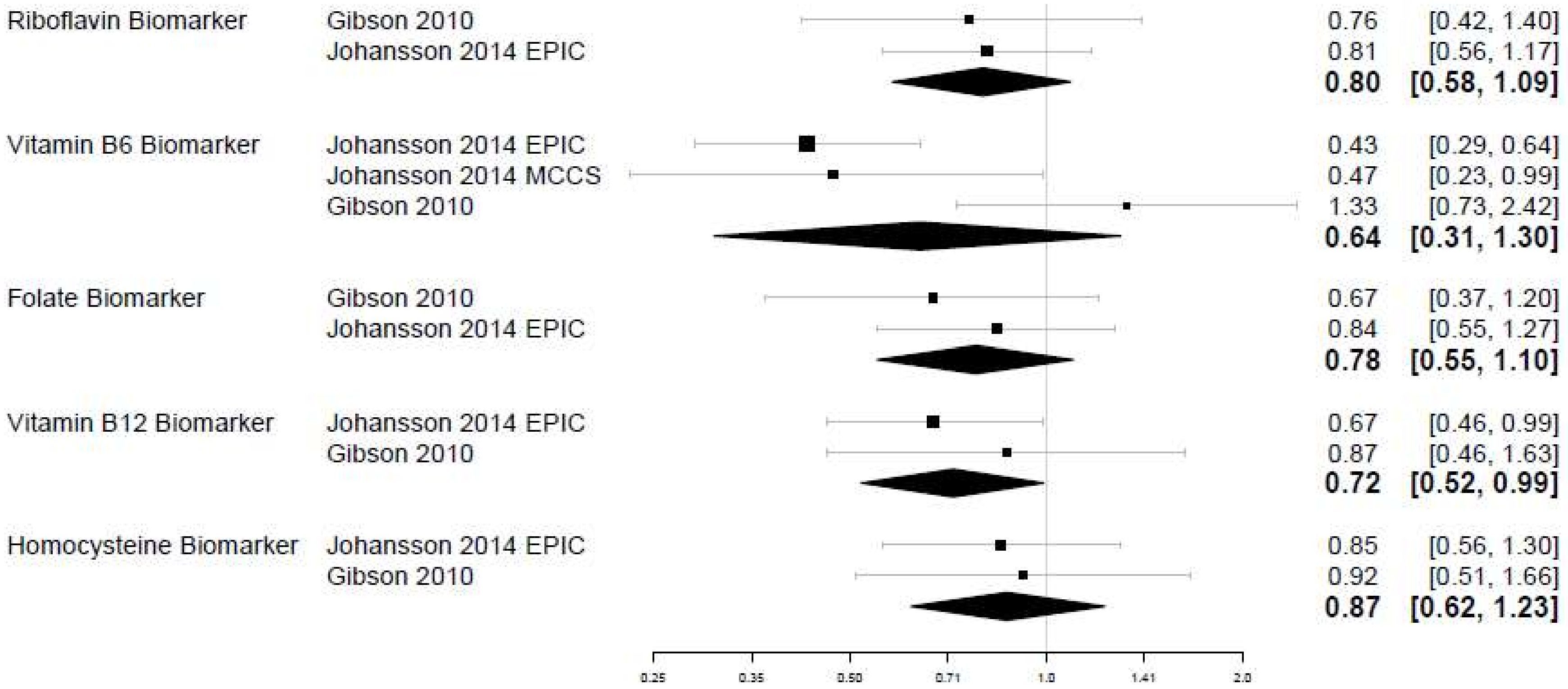

Online Resource 3: Forest plots for biomarker status exposures and RCC risk using a frequentist random-effects model.

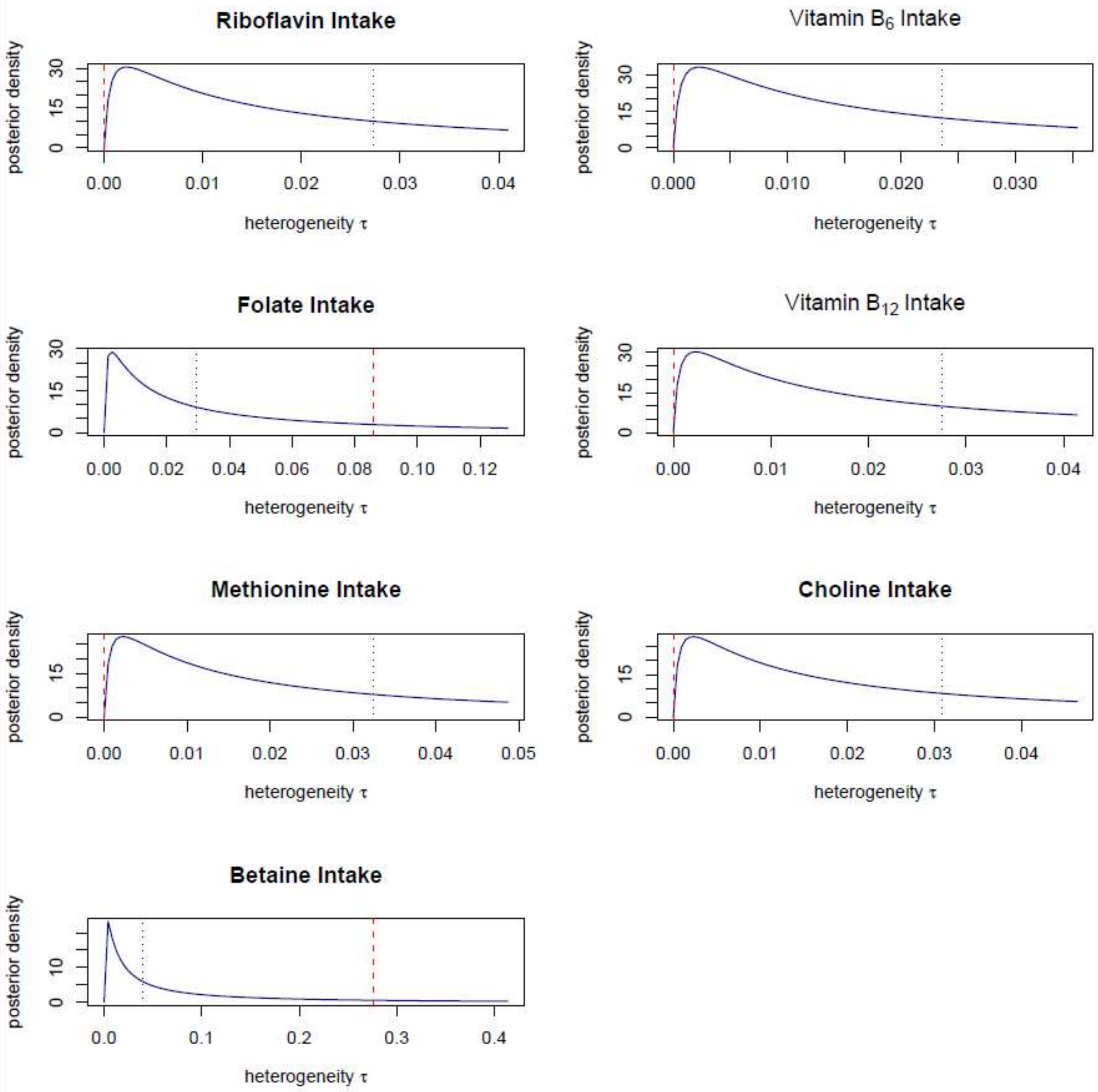

Online Resource 4: Estimates of τ for dietary intake exposures: Posterior distributions (blue solid lines) and medians (blue dotted lines) from the Bayesian model and point estimate (red dashed lines) from the frequentist random-effects model. Note that the scales on both axes vary between plots.

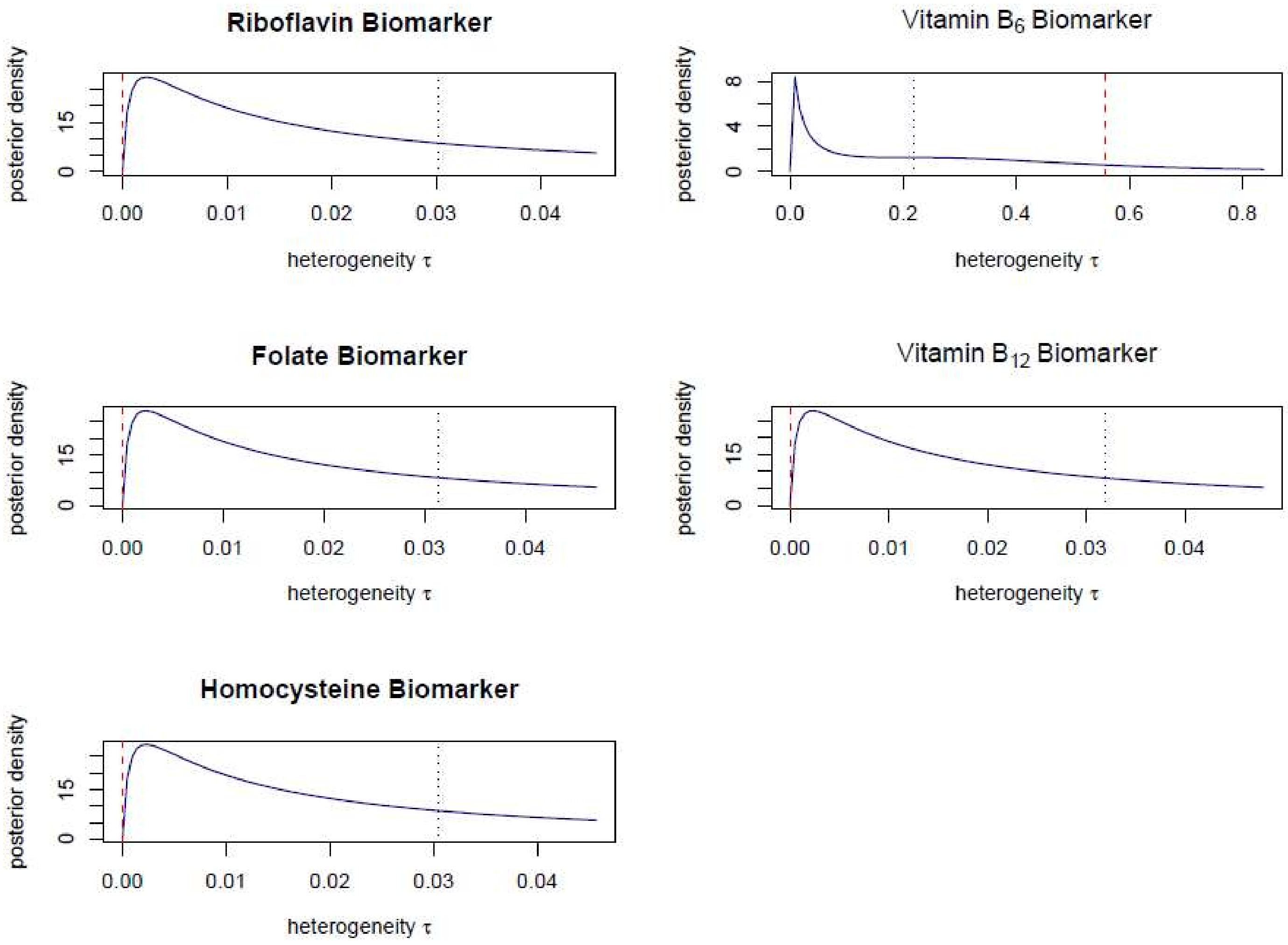

Online Resource 5: Estimates of τ for biomarker status exposures: Posterior distributions (blue solid lines) and medians (blue dotted lines) from the Bayesian model and point estimate (red dashed lines) from the frequentist random-effects model. Note that the scales on both axes vary between plots.

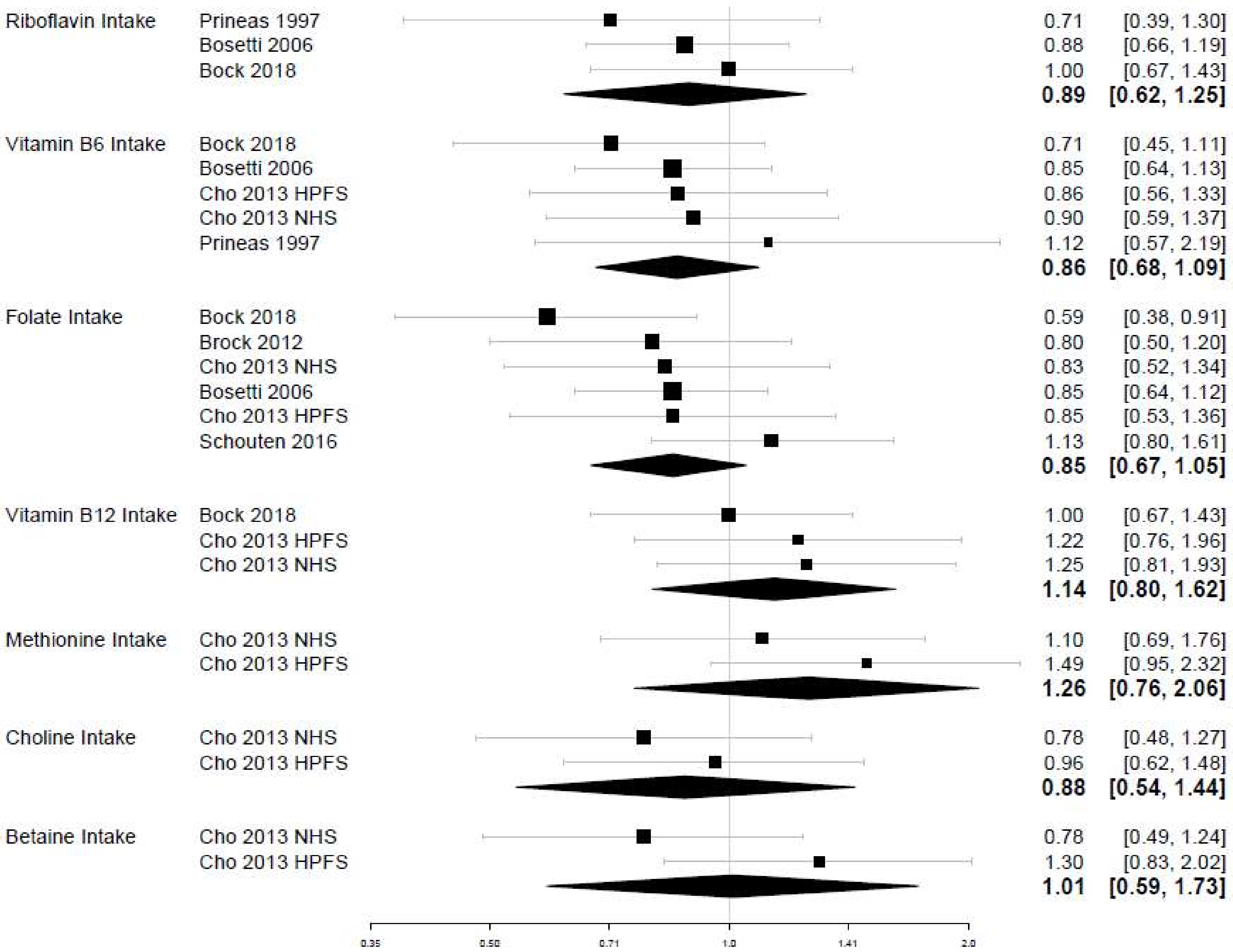

Online Resource 6: Forest plots for dietary intake exposures and RCC risk using a Bayesian model with theoretically informed prior distributions for μ and τ.

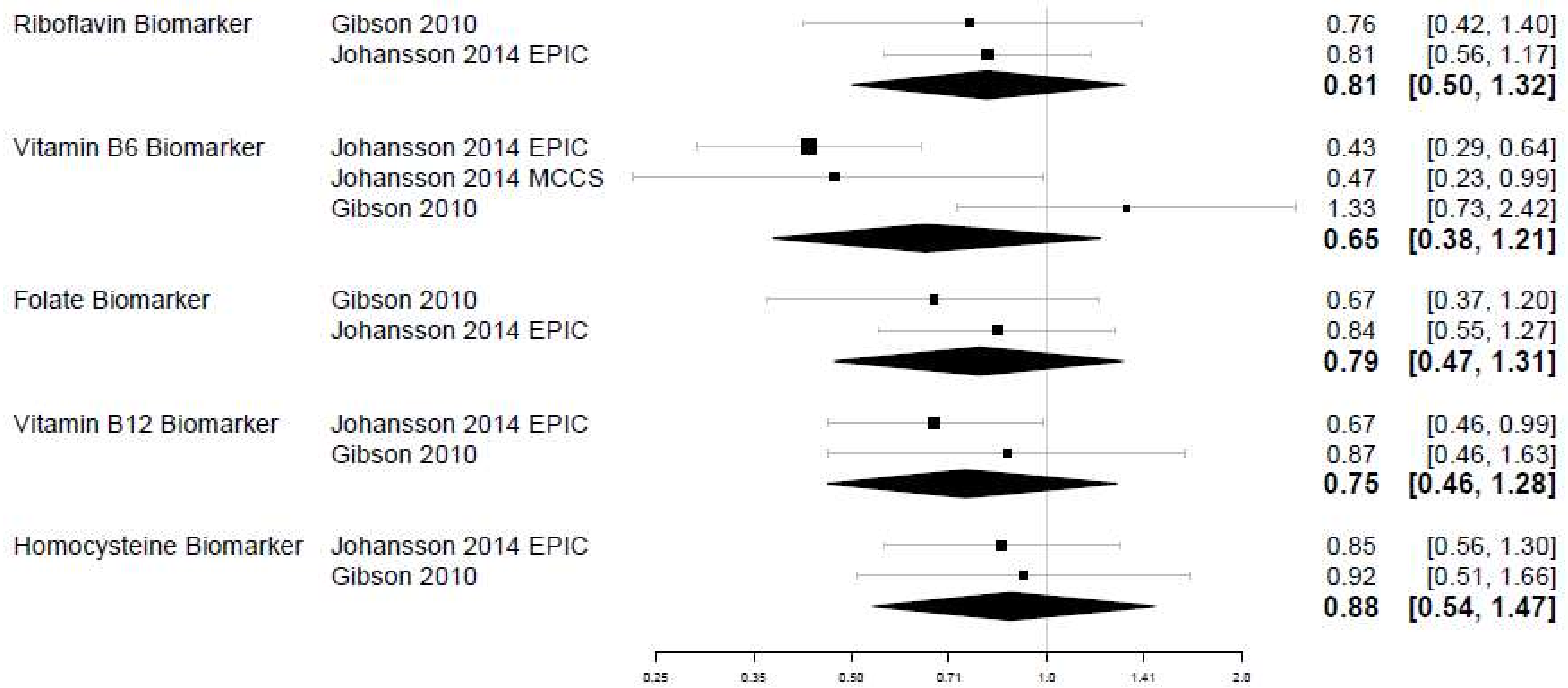

Online Resource 7: Forest plots for biomarker status exposures and RCC risk using a Bayesian model with theoretically informed prior distributions for μ and τ.

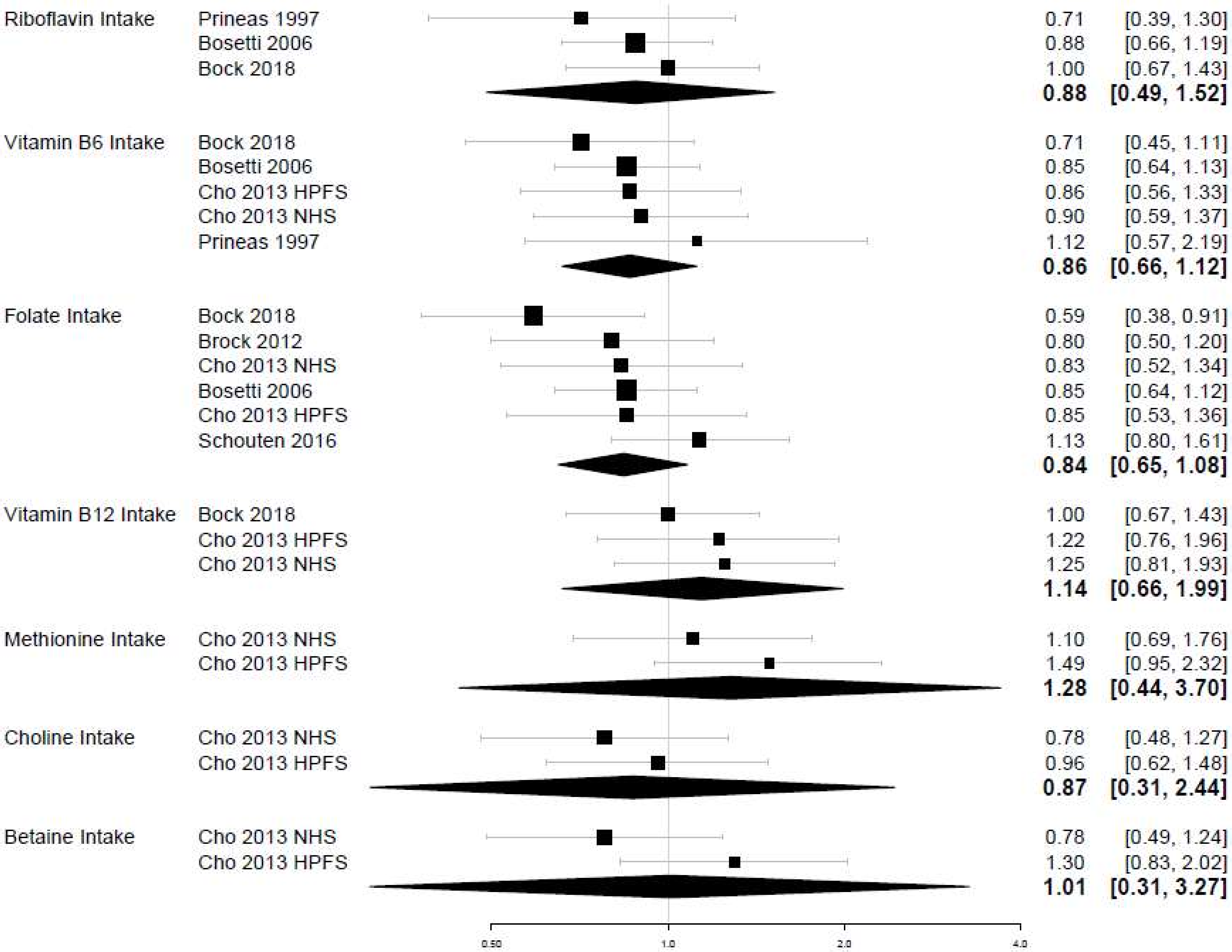

Online Resource 8: Forest plots for dietary intake exposures and RCC risk using a Bayesian model with weak prior distributions for μ and τ.

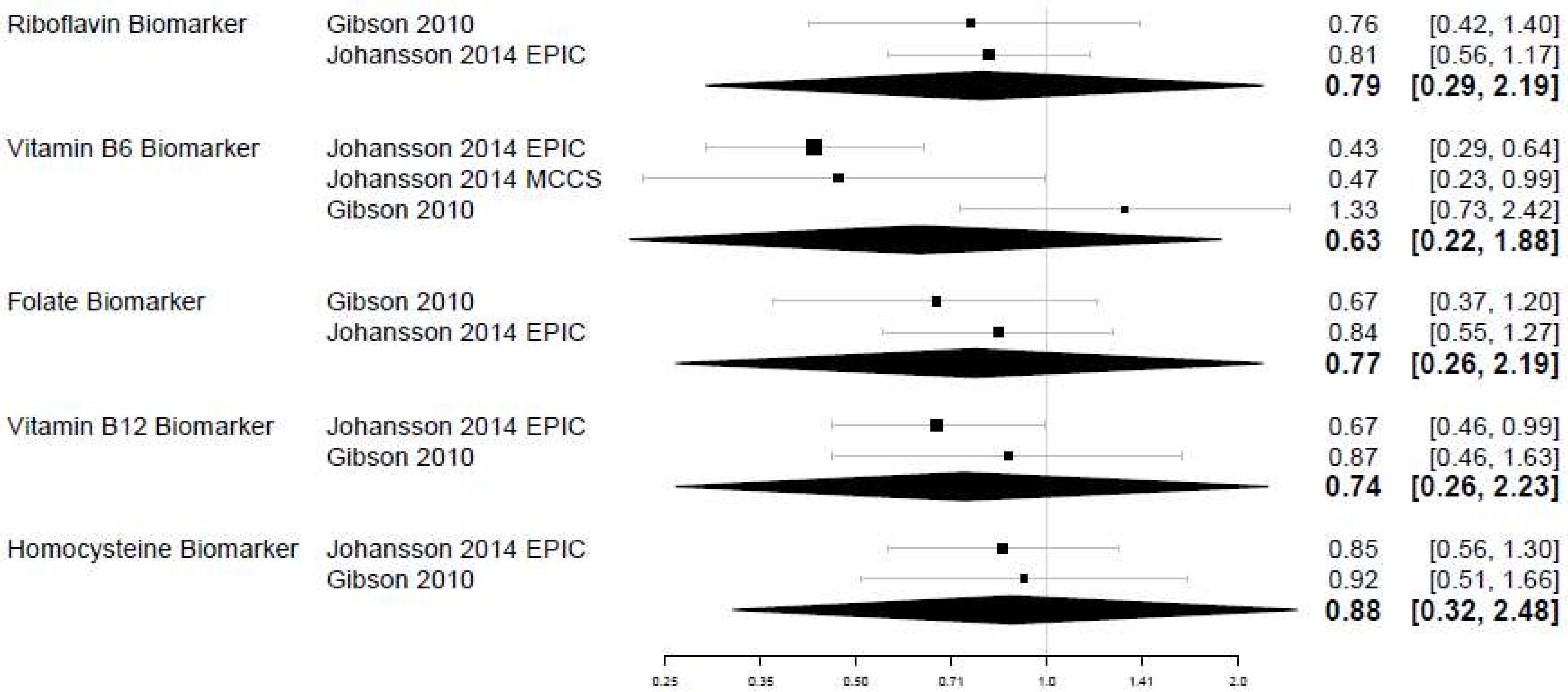

Online Resource 9: Forest plots for biomarker status exposures and RCC risk using a Bayesian model with weak prior distributions for μ and τ.

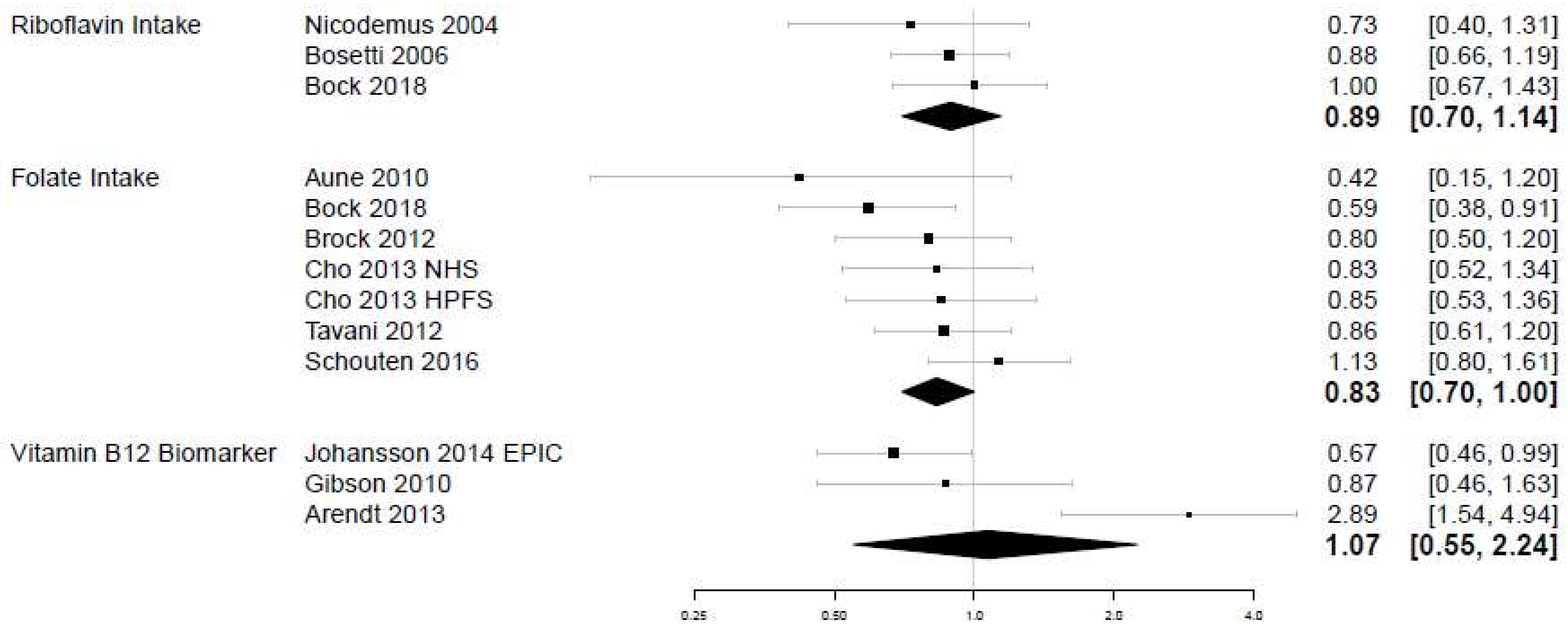

Online Resource 10: Forest plots with overall kidney cancer outcomes pooled with RCC-specific outcomes using the original Bayesian model.

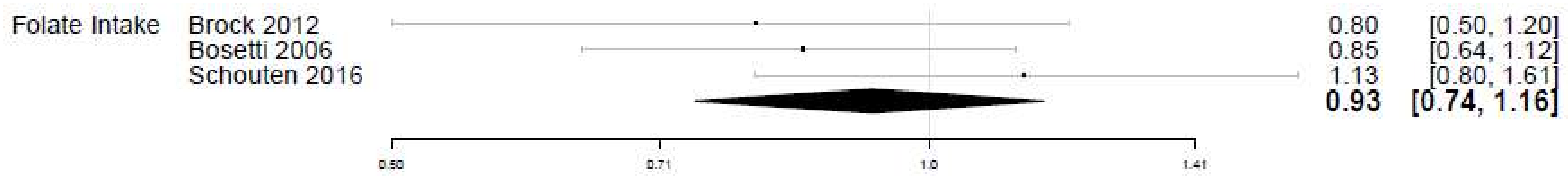

Online Resource 11: Forest plots including only studies with a modified NOS score of at least seven. Plots not shown for exposures which already met the criterion in original analyses.

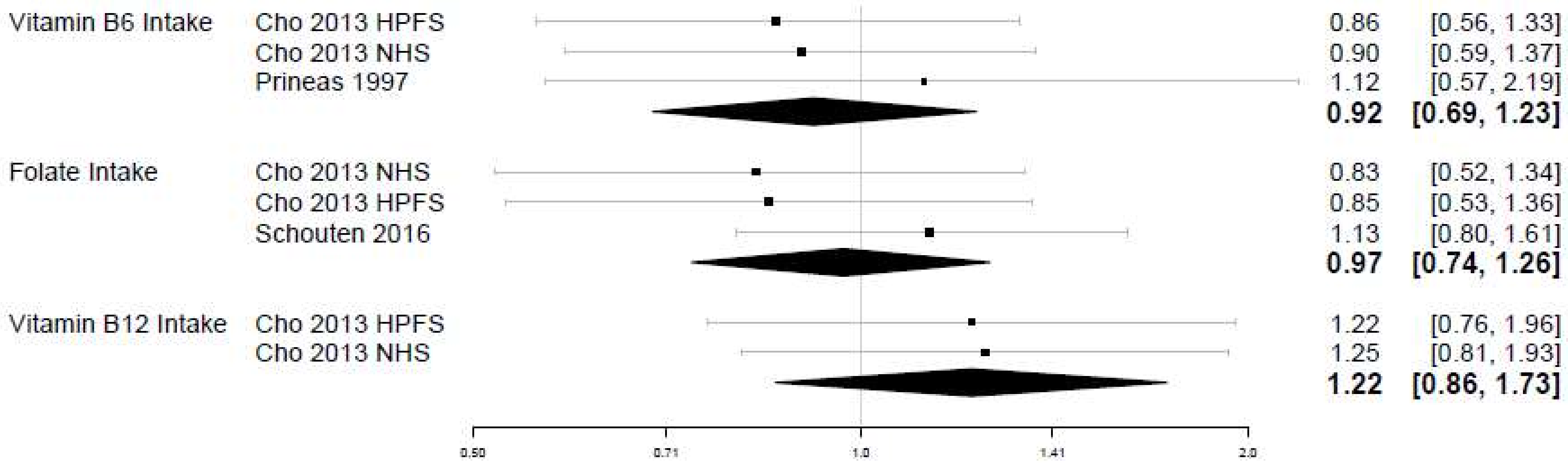

Online Resource 12: Forest plots for dietary intake exposures including only studies with prospectively collected dietary data. Plots not shown for exposures which already met the criterion in original analyses.

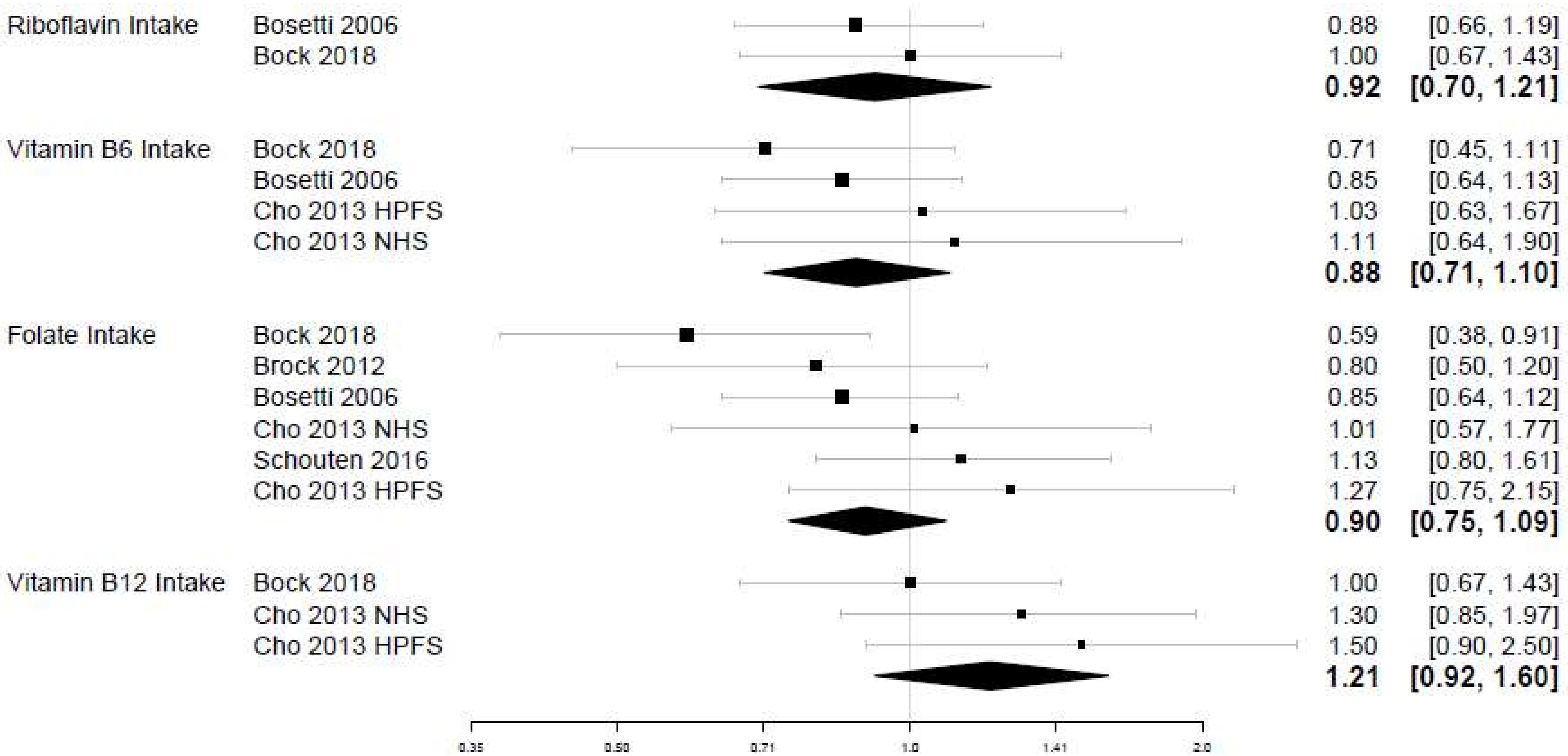

Online Resource 13: Forest plots for dietary intake exposures including only estimates from food consumption, excluding supplement use. Plots not shown for exposures which already met the criterion in original analyses.

Components of one-carbon metabolism and renal cell carcinoma: A systematic review and meta-analysis

European Journal of Nutrition

Joanna L Clasen, Alicia K Heath, Ghislaine Scelo, David C Muller

Department of Epidemiology and Biostatistics, School of Public Health, Imperial College London, London, UK
david.muller@imperial.ac.uk
